# Human serum albumin as a copper source for anticancer thiosemicarbazones

**DOI:** 10.1093/mtomcs/mfad046

**Published:** 2023-07-28

**Authors:** Martin Schaier, Enrico Falcone, Tomas Prstek, Bertrand Vileno, Sonja Hager, Bernhard K Keppler, Petra Heffeter, Gunda Koellensperger, Peter Faller, Christian R Kowol

**Affiliations:** Institute of Analytical Chemistry, Faculty of Chemistry, University of Vienna, Waehringer Str. 38, A-1090 Vienna, Austria; Vienna Doctoral School in Chemistry (DoSChem), University of Vienna, Waehringer Str. 42, A-1090 Vienna, Austria; Institut de Chimie, UMR 7177, CNRS, Université de Strasbourg, 4 Rue Blaise Pascal, 67000 Strasbourg, France; Institute of Analytical Chemistry, Faculty of Chemistry, University of Vienna, Waehringer Str. 38, A-1090 Vienna, Austria; Institut de Chimie, UMR 7177, CNRS, Université de Strasbourg, 4 Rue Blaise Pascal, 67000 Strasbourg, France; Center for Cancer Research and Comprehensive Cancer Center, Medical University of Vienna, Borschkegasse 8a, A-1090 Vienna, Austria; Institute of Food Chemistry and Toxicology, Faculty of Chemistry, University of Vienna, Waehringer Str. 38, A-1090 Vienna, Austria; Institute of Inorganic Chemistry, Faculty of Chemistry, University of Vienna, Waehringer Str. 42, A-1090 Vienna, Austria; Research Cluster ‘Translational Cancer Therapy Research’, A-1090 Vienna, Austria; Center for Cancer Research and Comprehensive Cancer Center, Medical University of Vienna, Borschkegasse 8a, A-1090 Vienna, Austria; Research Cluster ‘Translational Cancer Therapy Research’, A-1090 Vienna, Austria; Institute of Analytical Chemistry, Faculty of Chemistry, University of Vienna, Waehringer Str. 38, A-1090 Vienna, Austria; Research Cluster ‘Translational Cancer Therapy Research’, A-1090 Vienna, Austria; Institut de Chimie, UMR 7177, CNRS, Université de Strasbourg, 4 Rue Blaise Pascal, 67000 Strasbourg, France; Institut Universitaire de France (IUF), 1 rue Descartes, 75231 Paris, France; Institute of Inorganic Chemistry, Faculty of Chemistry, University of Vienna, Waehringer Str. 42, A-1090 Vienna, Austria; Research Cluster ‘Translational Cancer Therapy Research’, A-1090 Vienna, Austria

**Keywords:** serum proteins, albumin, thiosemicarbazones, copper, iron, ICP-MS

## Abstract

Thiosemicarbazones (TSCs) are a class of biologically active compounds with promising anticancer activity. Their typical mechanism, especially of the clinically far developed representative Triapine, is chelation of iron (Fe), with the Fe-containing enzyme ribonucleotide reductase as primary intracellular target. However, for the subclass of terminally disubstituted, nanomolar-active derivatives like Dp44mT and Me_2_NNMe_2_, recent findings suggest that the chelation, stability, and reduction properties of the copper(II) (Cu) complexes are essential for their modes of action. Consequently, it is important to elucidate whether blood serum Cu(II) is a potential metal source for these TSCs. To gain more insights, the interaction of Triapine, Dp44mT or Me_2_NNMe_2_ with purified human serum albumin (HSA) as the main pool of labile Cu(II) was investigated by UV-vis and electron paramagnetic resonance measurements. Subsequently, a size-exclusion chromatography inductively coupled plasma mass spectrometry method for the differentiation of Cu species in serum was developed, especially separating the non-labile Cu enzyme ceruloplasmin from HSA. The results indicate that the TSCs specifically chelate copper from the *N*-terminal Cu-binding site of HSA. Furthermore, the Cu(II)-TSC complexes were shown to form ternary HSA conjugates, most likely via histidine. Noteworthy, Fe-chelation from transferrin was not overserved, even not for Triapine. In summary, the labile Cu pool of HSA is a potential source for Cu-TSC complex formation and, consequently, distinctly influences the anticancer activity and pharmacological behavior of TSCs.

## Graphical Abstract

**Graphical Abstract:**
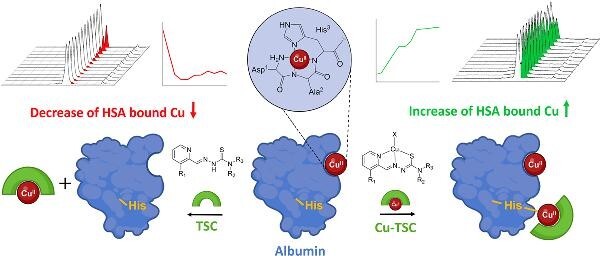
Interaction of Cu(II)-HSA and thiosemicarbazones with formation of Cu(II)-TSC and its ternary complex with HSA.

## Introduction

**Scheme 1:**
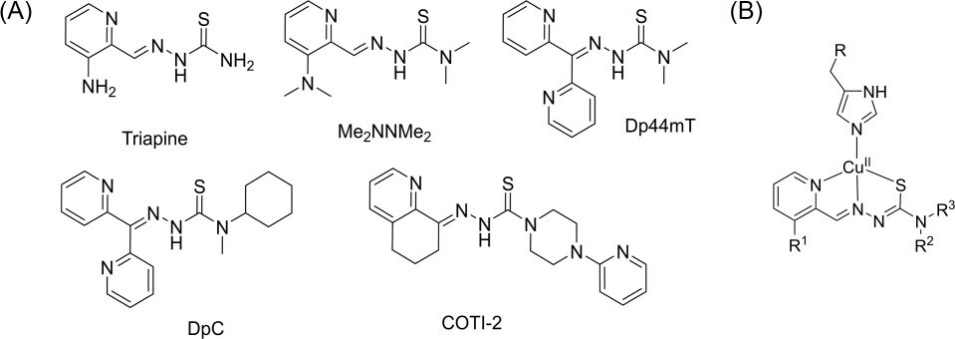
(A) Chemical structures of (clinically) investigated TSCs. (B) Ternary His-Cu(II)-TSC complex (His = histidine).

Thiosemicarbazones (TSCs) are biologically active compounds that have been developed for the treatment of diverse diseases including cancer.^1^ Here, especially α-*N*-heterocyclic TSCs are in the focus of interest, as they are highly potent metal chelators. Consequently, drugs like Triapine (3-AP) or Dp44mT (Scheme 1) have been originally developed to exploit the enhanced need of cancer cells for iron (Fe)^2,3^ with the Fe-containing enzyme ribonucleotide reductase as proposed intracellular target.^4^ Based on its promising anticancer activity, Triapine was not only tested in >35 phase I and II clinical trials^5–9^ but also entered recently a phase III study against cervical or vaginal cancer patients in combination with cisplatin and radiation therapy (study number NCT02466971; www.clinicaltrials.gov).^10^ Moreover, also two other TSC derivatives, di-2-pyridyl ketone 4-cyclohexyl-4-methyl-3-thiosemicarbazone (DpC, study number NCT02688101) and 4-(pyridine-2-yl)-N-([(8E)-5,6,7,8-tetrahydroquinolin-8-ylidene]amino))piperazine-1-carbothioamide (COTI-2, study number NCT02433626) started clinical phase I evaluation (Scheme 1). Noteworthy, these two compounds as well as Dp44mT (the predecessor of DpC) and a dimethylated Triapine derivative (Me_2_NNMe_2_) developed in our laboratory represent a subclass of TSCs characterized by a ∼500-fold higher anticancer activity compared to Triapine in cell culture.^11,12^ Several studies suggested that this enhanced efficiency could be based on an additional mode-of-action, associated with their ability to chelate also other essential metal ions beside Fe, especially copper (Cu).^13–15^ Indeed, we were recently able to show that the anticancer activity of TSCs is correlated with the stability and, especially, reduction kinetics of their Cu complexes.^16^ Moreover, this also influenced the resistance profile of cancer cells, as TSCs with high affinity for Cu form ternary complexes with glutathione (GSH), rendering them substrates for the efflux pump ATP-binding cassette transporter C1 (ABCC1).^17,18^ Noteworthy, ABCC1 is also expressed in tissues important for absorption, metabolism, and elimination (liver and kidney),^19^ which could also indicate an impact on the pharmacology of the respective TSCs. However, so far it is unclear whether and where TSCs can bind Cu in the body. In general, several distinct proteins (e.g. Ctr1, Atox1, CCS, and ATP7A/B) tightly control the homeostasis of Cu ions in humans.^20^ In fact, Cu is always found in protein-bound form, and it is widely accepted that under healthy conditions, there is basically no free Cu available (in the circulation).^21^ Moreover, Cu outside the cells is usually present as Cu(II), while intracellularly it is mainly Cu(I).^21^ However, TSCs are strong Cu(II) chelators, while their planar structure is not suited for efficient Cu(I) binding. Consequently, we hypothesized that TSCs interact especially with Cu of extracellular origin.

There are two main Cu carrier proteins in human serum: Ceruloplasmin and human serum albumin (HSA). While in the case of ceruloplasmin, the six Cu sites are buried inside the protein and, hence, are not accessible for external chelators, Cu on HSA can be chelated from molecules having a suitable affinity.^21^ In particular, HSA physiologically binds ∼3 µM Cu(II) via a well-characterized *N*-terminal tripeptide (ATCUN) motif with a dissociation constant of *K*_d_ ∼10^−^^13^ at pH 7.4.^22^ Of note, less than 1% HSA is normally loaded with Cu(II).^21^ In contrast to Cu, serum Fe exists as Fe(III) almost exclusively bound to transferrin with a very high affinity and a dissociation constant of *K*_d_ ∼ 10^−22^ at pH 7.4.^23^ Under healthy conditions, transferrin is only loaded to a third with Fe(III). Hence, about 20 µM transferrin with two unoccupied Fe(III)-binding sites are present in blood plasma.^24^ As TSCs have a lower Fe(III) affinity than transferrin,^25,26^ they are not expected to be able to withdraw Fe(III) from transferrin (this situation changes inside the cells, where Fe is weakly bound, and this labile Fe pool is mainly in the reduced Fe(II) state^27^). Consequently, the present study focuses on Cu(II) and the investigation whether or not different TSCs (Triapine, Dp44mT, and Me_2_NNMe_2_) are able to form a complex with Cu(II) in blood plasma. Towards this aim, UV-vis and electron paramagnetic resonance (EPR) measurements with HSA, as the main labile Cu(II) pool, were conducted. Furthermore, a size-exclusion chromatography inductively coupled plasma mass spectrometry (SEC-ICP-MS) method was developed to analyze the interaction in plasma.

## Results

### Interaction of TSCs with Cu and HSA

**Fig. 1:**
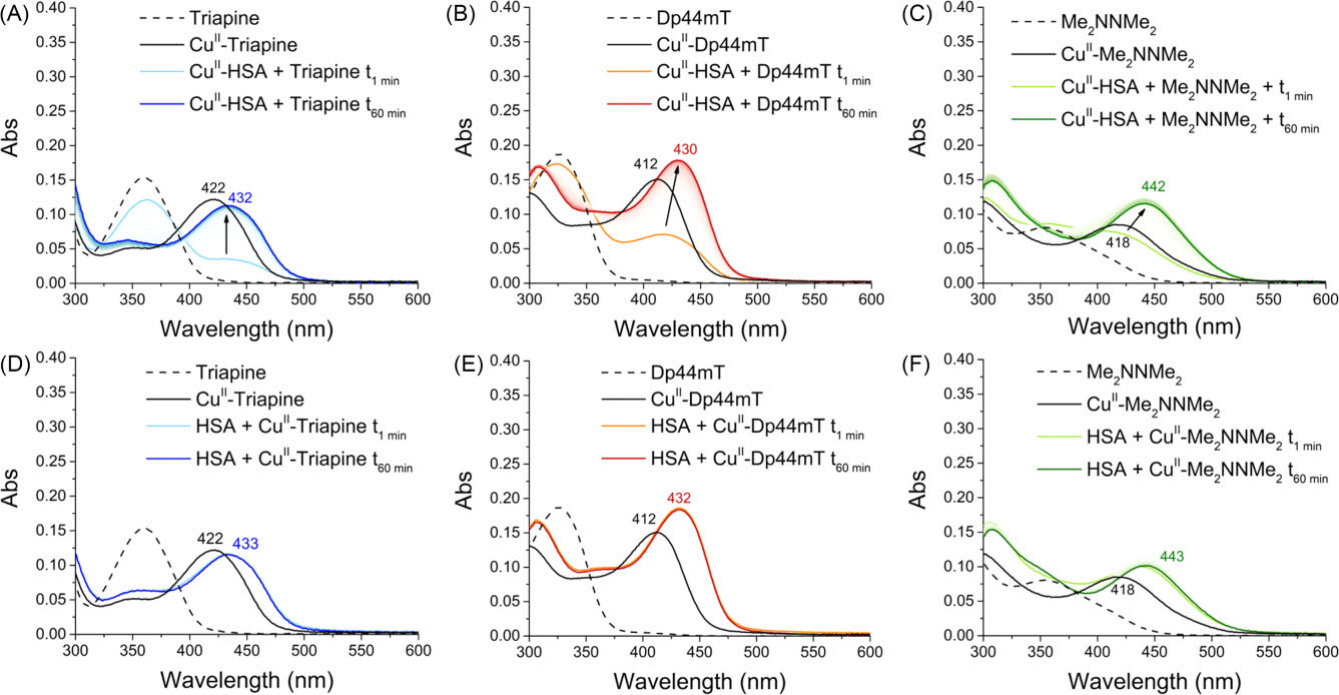
Competition of TSCs and HSA for Cu(II) (A, B, C) and interaction of pre-formed Cu(II)-TSCs with HSA (D, E, F). (A, D) Triapine (blue); (B, E) Dp44mT (red); (C, F) Me_2_NNMe_2_ (green). Conditions: [TSC] = 10 µM, [Cu(II)] = 10 µM, [HSA] = 100 µM, HEPES 500 mM pH 7.4 (DMSO 1%). T = 37°C. Background absorption spectra of HSA or Cu(II)-HSA were subtracted.

The ability of Triapine, Dp44mT, and Me_2_NNMe_2_ (10 µM) to compete with HSA for Cu(II) was assessed by incubating the ligands with the pre-formed Cu(II)-HSA (1:10 Cu: HSA; 100 µM HSA) complex at 37°C. Initially, DpC and COTI-2 were also included; however, their low solubility (requiring ∼30% DMSO) prevented meaningful measurements in the presence of HSA. The formation of Cu(II)-TSCs complexes was first monitored via UV-vis absorption spectroscopy, possible due to the characteristic thiolate-to-metal charge-transfer bands arising upon metal binding (Table 1 and Fig. 1). In contrast, the Cu(II)-HSA complex does not show absorption bands in this spectral region (note that the intensity of d–d bands is too low to be detectable at this concentration).

**Table 1.** Absorption wavelength (nm) and extinction coefficient (M^−1^cm^−1^, in brackets) of TSCs and their mixture with HSA and Cu ions

	Triapine	Dp44mT	Me_2_NNMe_2_
TSC	360 (15 300)	326 (18 700)	364 (8050)
TSC-Cu	422 (12 200)	412 (15 100)	418 (8460)
TSC-Cu-HSA	432 (11 600)*	430 (18 600)*	442 (10 120)*
TSC-HSA	437 (n/a)	417 (n/a)	442 (n/a)

*extinction coefficients were calculated assuming that Cu-TSC is completely bound to HSA forming a single species.

After addition of the TSCs, new bands (Fig. 1; light blue, orange and light green lines) appeared and increased over time (dark blue, red, and dark green lines), commensurate with the Cu(II) transfer from HSA to TSCs. Moreover, these bands were red-shifted compared to those of the Cu(II)-TSC complexes in absence of HSA (Table 1 and Fig. 1A–C, black lines), suggesting the formation of ternary TSC-Cu(II)-HSA complexes. The same bands were observed, when the pre-formed Cu(II)-TSC complexes were added to HSA (Fig. 1D–F). As a control, since metal-independent hydrophobic interactions can also occur between TSCs and HSA,^28^ we recorded UV-vis spectra of the TSC-HSA mixtures in the absence of Cu(II) (Fig. S1). These data showed a decrease of the TSC bands upon addition to HSA and the concomitant appearance of red-shifted bands above 400 nm (Fig. S1), suggesting an interaction of all TSCs with HSA, although to a different extent (Fig. S1). Of note, the same band (at about 442 nm) appeared for the mixture of Me_2_NNMe_2_ and HSA in the absence and presence of Cu(II), questioning the attribution of this band to a direct Cu(II)-Me_2_NNMe_2_ interaction.

These findings are consistent with the literature, as some TSCs and Cu-TSC complexes have been shown to bind to the drug-binding sites in HSA (notably in subdomain IB and IIA) and also form Cu-bridged ternary complexes through His residues in HSA (e.g. His146 in subdomain IB and His242 in subdomain IIA).^28–31^ Remarkably, considering that imidazole groups have generally only millimolar affinity for Cu complexes with tridentate ligands (e.g. GHK peptide^32^), the interactions between the ligand and hydrophobic pockets within HSA appear to enhance the affinity between the protein and the Cu-TSC.

**Fig. 2:**
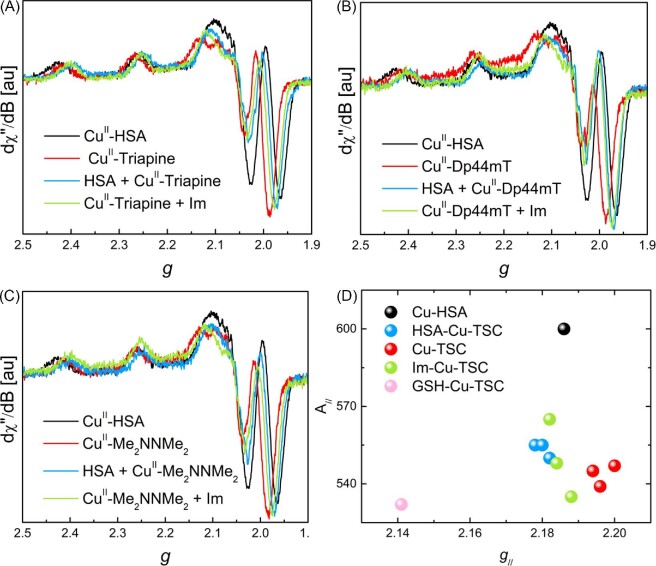
EPR spectra (A, B, C) and Peisach–Blumberg plot (D) of Cu(II)-HSA (black), Cu(II)-TSCs (red), HSA/Cu(II)-TSC mixtures (blue), TSC-Cu(II)-Im ternary complexes (green), and GSH-Cu(II)-Dp44mt (pink) Conditions: [TSC] = 300 µM, [Cu(II)] = 250 µM, [HSA] = 300 µM, [Im] = 300 µM, [GSH] = 300 µM, HEPES 50 mM pH 7.4 (DMSO ∼5%), glycerol 10% (v/v), T = 100 K. TSC = Triapine, Dp44mT or Me_2_NNMe_2_; Im = imidazole.

**Fig. 3:**
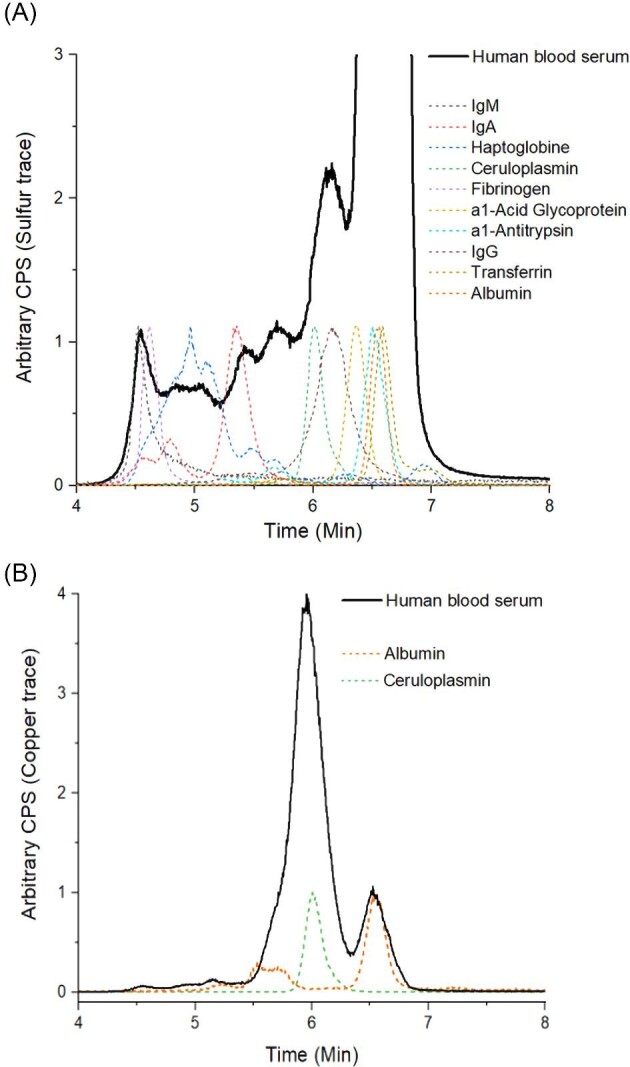
(A) Overlay plot of the high-molecular weight SEC-ICP-MS sulfur trace of human blood serum (black solid line) with 10 protein standards (dotted lines). (B) Overlay plot of the ICP-MS Cu trace of human blood serum (black solid line) with HSA and ceruloplasmin (dotted lines).

In order to further assess the formation of Cu(II)-bridged ternary complexes between the TSCs and HSA, we used EPR spectroscopy, which is sensitive to the Cu(II) coordination sphere and not influenced by ligand-protein interactions. Thus, continuous-wave (CW) X-band EPR spectra of Cu(II)-TSCs, Cu(II)-HSA and pre-formed Cu(II)-TSCs added to HSA were recorded at 100 K (Fig. 2A–C) and simulated to obtain the characteristic *g*_//_ and A_//_ parameters for each complex (Table S1). The spectra of the ternary HSA/Cu(II)-TSC mixtures show one set of EPR signals with lower *g*_//_ values compared to both Cu(II)-HSA and Cu(II)-TSCs, suggesting the presence of a distinct major species (rather than a mixture of the two binary species). To better compare the spectra, each pair of *g*_//_ and A_//_ parameters was represented in a Peisach-Blumberg plot (Fig. 2D). This plot clearly shows that Cu(II) coordination in HSA/Cu(II)-TSC mixtures (blue dots) is different from that of Cu(II)-HSA (black dot), and closer to that of free Cu(II)-TSCs alone (red dots). The similarity between the spectra of the ternary mixture with those of Cu(II)-TSCs confirms that Cu(II) is basically coordinated to TSCs even in the presence of HSA, while the shift towards lower *g*_//_ observed in the presence of HSA is consistent with the formation of a ternary HSA-Cu(II)-TSC complex through the replacement of a fourth oxygen donor (e.g. H_2_O or DMSO) with an imidazole ligand provided by HSA (Scheme 1). In fact, similar downshifts of the *g*_//_ values are observed upon addition of imidazole (Im) to the samples containing Cu(II)-TSCs (Fig. 3D, green dots). Of note, the binding of HSA to Cu(II)-TSCs via its unique reduced cysteine 34 residue can be ruled out by comparing the EPR parameters of the HSA/Cu(II)-TSC mixtures with the ternary complex between Cu(II)-Dp44mT and GSH, a Cys-containing tripeptide (Fig. 2D, pink dot). Finally, it is worth noting that due to the significant signal-to-noise ratio, the presence of minor Cu(II)-HSA and especially Cu(II)-TSC species within the ternary mixtures cannot be ruled out. In fact, it is likely that HSA-bound and unbound Cu(II)-TSC co-exist.

When analyzing the data from Figs. 1 and 2, all the metal-free TSCs revealed to be able to remove Cu from a 10-fold excess of HSA and to form ternary complexes with the His residues of the protein.

All investigations so far have been performed in buffered aqueous solutions containing only HSA. As a next step, we wanted to analyze the behavior in a much more complex system like human serum. Unfortunately, in such biological concentrations and matrices UV-vis or EPR measurements are hardly feasible. Therefore, we established an SEC-ICP-MS method to analyze ^32^S, ^63^Cu, and ^56^Fe levels in human serum upon addition of TSCs and their Cu complexes.

### SEC-ICP-MS method establishment

A rapid UPLC-type size exclusion chromatographic method was developed entailing low metal backgrounds and fit for purpose, for time-series measurements without compromising on separation efficiency. Four different stationary phases were compared, differing in particle size, pore size, length, and column chemistry upon monitoring the ^32^S (^32^S^16^O), ^56^Fe (^56^Fe^16^O), and ^63^Cu traces in human serum (Fig. S2). A particular focus was laid on the separation of ceruloplasmin from HSA. The column utilizing ethylene-bridged hybrid (BEH)-based particle technology (Acquity UPLC Protein BEH 200 Å) showed the best performance (Fig. S2). In addition, the elemental background was comparable to the PEEK column (MabPac SEC-1 300 Å) despite the steel housing (Fig. S3). The overall separation time could be reduced to 12 min.

### Identification of serum proteins

Association of elemental signals to plasma proteins was accomplished by retention time matching with standards. The 10 most abundant plasma proteins (except α2-macroglobulin) and ceruloplasmin were analyzed with the new SEC-ICP-MS method. The used protein standards were HSA (100 µM), IgG (100 µM), fibrinogen (100 µM), IgA (10 µM), transferrin (100 µM), α1-antitrypsin (100 µM), IgM (1,1 µM), haptoglobin (30 µM), α1-acid glycoprotein (100 µM), and ceruloplasmin (40 µM). Retention times were assessed based on the sulfur trace, as all proteins contain the amino acids Cys and methionine (Met) (Fig. 3A; for the whole range; see Fig. S4). The major serum protein HSA (6.6 min; 67 kDa and the HSA dimer at 5.7 min) coelutes at least with three other proteins, namely transferrin (80 kDa), α1-acid-glycoprotein (40 kDa), and α1-antitrypsin (52 kDa). The signal of the second most abundant protein IgG (∼150 kDa) nicely matched a peak at ∼6.2 min in whole serum. IgA (160 kDa) eluted at around 5.4 min, haptoglobin in a broad peak at around 5 min, fibrinogen (∼340 kDa) at 4.6 min, and pentameric IgM (up to ∼900 kDa) at 4.5 min. Although the hydrodynamic radius of the proteins is the decisive parameter for SEC, there was a good correlation between elution time and protein mass.

**Fig. 4:**
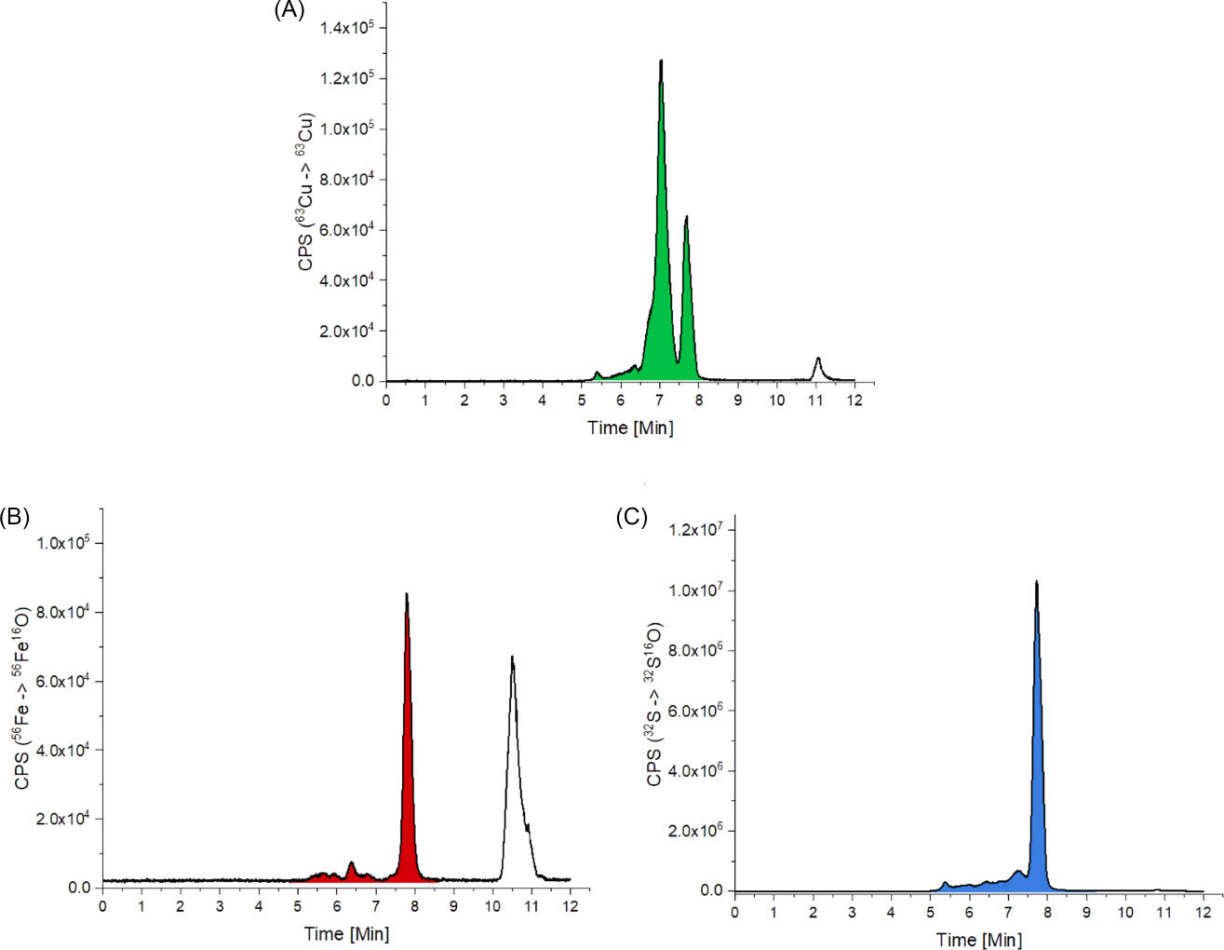
(A) ^63^Cu, (B) ^56^Fe, and (C) ^32^S traces of human serum (peak integration areas are colored). To increase the reproducibility of the measurements a lower flow rate was used, which resulting in a higher retention time shift of ∼1.1 min for all peaks compared to the reference measurements in Fig. 3.

In addition to ^32^S, ^56^Fe and ^63^Cu were also measured for human serum (Fig. 4) and for all protein standards (Fig. S5). Noteworthy, to increase the reproducibility of the measurements, a lower flow rate was used which resulted in a higher retention time shift of ∼1.1 min for all peaks compared to the reference measurements in Fig. 3. The ^63^Cu trace (Fig. 4A) indicated two main peaks at 7.0 and 7.7 min, which nicely fitted the retention time of ceruloplasmin and HSA (considering the time shift of 1.1 min) and confirmed that these two proteins contribute most to the Cu content in human serum (Fig. 3B). In the case of ^56^Fe, a major peak was observed again at 7.7 min (containing transferrin) and in the low-molecular weight range a large signal at 10.5 min (Fig. 4B). At the same retention time no signal was visible in the ^32^S trace (Fig. 4C), at which usually metal-peptide, metal-amino acid, and other low-molecular species are expected. Therefore, the ^56^Fe peak at 10.5 min can most likely be attributed to heme (which does not contain any sulfur). The presence of heme in serum is common, as erythrocytes can burst during the serum extraction process from blood, thereby releasing heme from hemoglobin (hemolysis).

Repeated injections of human serum over 120 min showed excellent intermediate precision of <3% (see Fig. S6) considering the sum peak areas for ^32^S, ^56^Fe, and ^63^Cu, respectively, representing the high-molecular weight fraction (as coloured in Fig. 4), which was key for the planned incubation studies. Analysis of TSC and their Cu complexes is known to be challenged by material interactions along the chromatographic separation process causing memory effects, peak tailing, and general extra-column broadening. In order to exclude such unwanted interactions, the elution behaviour of the TSCs and their Cu complexes was studied via flow injection analysis. All compounds showed acceptable elution profiles, albeit the peak shapes were distinctly broader compared to a standard solution of Cu(NO_3_)_2_ (Fig. S7 for Cu-Triapine). Finally, the quantification of the Cu content after injection of Cu-TSCs was in good agreement with the theory, confirming quantitative elution (a blank run after each compound was applied).

### Spiking metal-free TSCs and their Cu complexes into serum

**Fig. 5:**
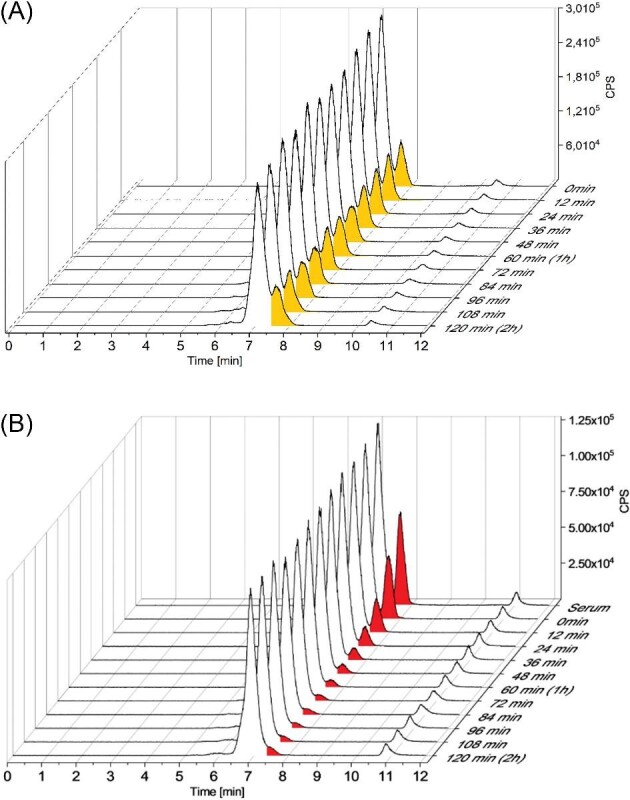
Cu-ICP-MS of serum (A) untreated or (B) spiked with 10 µM of metal-free Dp44mT.

Serum was incubated with all three metal-free TSCs at different concentrations (1, 10, and 100 µM) for 2 h. In agreement with the stability measurements (Fig. S6), no visible change could be observed in the ^63^Cu trace of just serum within 120 min. However, after the addition of the TSCs, a distinct time-dependent decrease of the ^63^Cu levels in the HSA peak at 7.7 min was detected (Fig. 5 for Dp44mT).

**Fig. 6:**
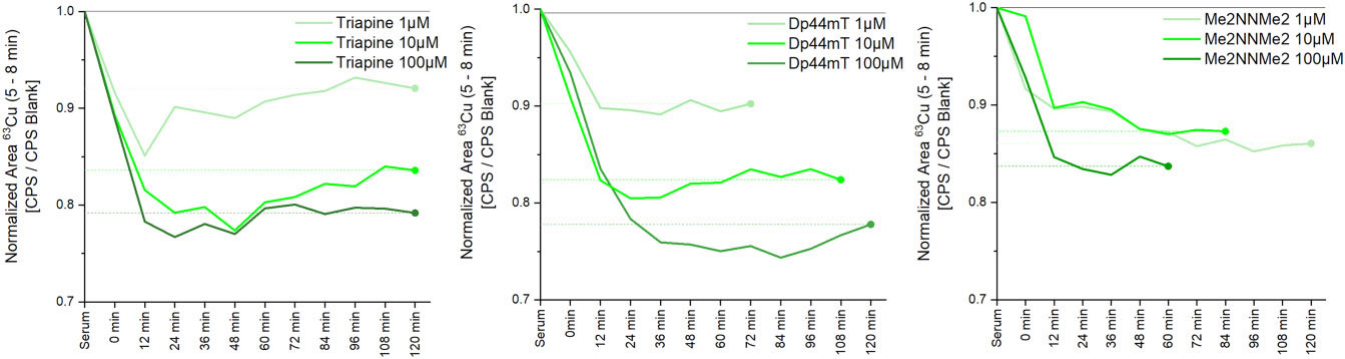
Cu levels (integration range from 5.0–8.0 min) of serum incubated with the three different metal-free TSCs at 1 µM, 10 µM, and 100 µM concentration over 120 min. The stable Cu content from ceruloplasmin corresponds to ∼70% of the area.

In contrast, the Cu signal at 7 min, originating from ceruloplasmin, was stable for all TSCs (∼70% of the total Cu content). This is in good agreement with the availability of the Cu ions from these two proteins: HSA as a transporter is able to release Cu ions, whereas in the case of ceruloplasmin, the Cu ions are not exchangeable. Therefore, the addition of a metal-free TSC resulted in the release of Cu from HSA most likely via formation of a Cu-TSC complex. However, it has to be mentioned that this complex did not appear as a new signal in the chromatogram. As we focused on copper adducts of proteins, it was not possible to retrieve low molecular weight copper species or inorganic copper with these methods. This is a well-known disadvantage of element speciation analysis by SEC.^33^ When comparing the addition of Dp44mT with that of Triapine (Figs. 6 and S8), for the latter slightly higher Cu levels remain at the HSA after 120 min, which is in good agreement with the lower affinity of Triapine for Cu ions.^16^ In comparison, in the case of Me_2_NNMe_2_, unexpectedly less Cu was ‘removed’ although the Cu-complex stability constants (in pure water) are distinctly higher than for Triapine^16^ (see discussion below).

The ^56^Fe trace of the high-molecular weight fraction was widely unaffected by the addition of any of the metal-free TSCs (Fig. S9). The small changes observed were also visible in untreated serum. This confirms, as already mentioned above, that transferrin-bound Fe(III) is not accessible for the TSCs.

**Fig. 7:**
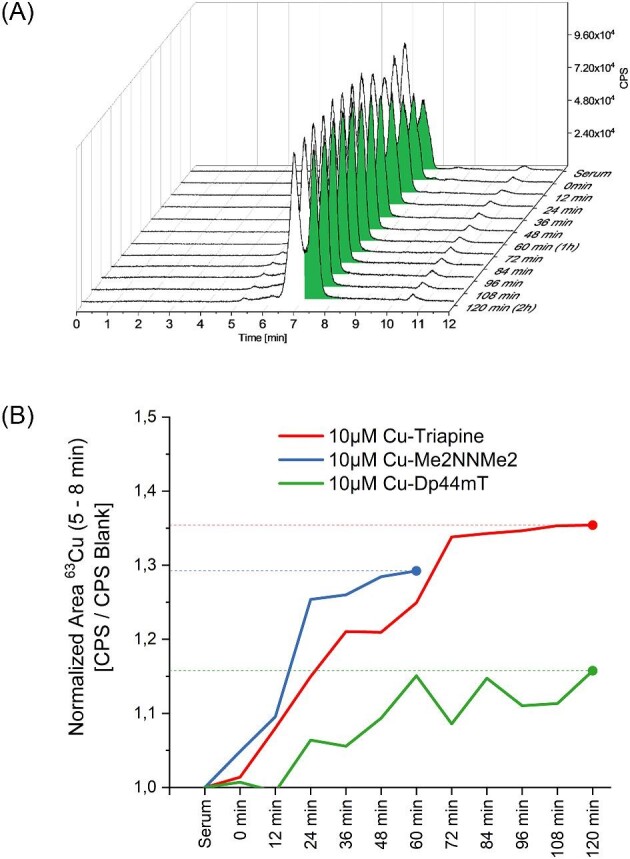
(A) Cu levels of serum treated with Cu-Triapine (10 µM). (B) Cu levels (integration range from 5.0–8.0 min) of serum incubated with the three different Cu-TSC complexes at 10 µM over 120 min.

Finally, the serum was incubated with all three Cu-TSC complexes at 10 µM for 2 h. In the case of Triapine, a distinct increase in the ^63^Cu trace could be observed (Fig. 7A). Again, the signal attributed to ceruloplasmin did not change. However, the levels of HSA-bound Cu steadily increased up to ∼ 1 h and then remained constant. Unfortunately, like in the measurements before, we did not see the free Cu-TSC complexes added. In the case of incubation with Cu-Me_2_NNMe_2_ and Cu-Triapine, the changes of the Cu-HSA levels were comparable, whereas Cu-Dp44mT showed lower binding (Figs. 7B and S10). The ^56^Fe trace was again widely unaffected by the addition of any of the Cu-TSCs (data not shown).

## Discussion

Already in the first comprehensive studies of α-*N*-heterocyclic TSCs as anticancer agents, the important role of metal ions in their mode of action was described.^34^ For a long time, especially Fe(II) and Fe(III) were in the focus of interest and for Triapine, currently studied in clinical phase III, Fe is still assumed to be crucial for its activity (and its main side effect methemoglobinemia).^13,35^ In general, complexation of TSCs with Cu(II) ions is mainly discussed for terminally di-alkylated TSCs such as Dp44mT, DpC, and Me_2_NNMe_2_, which are characterized by cytotoxic activities in cell culture in the nanomolar range. These compounds also show very strong synergistic activities with Cu(II) salts, in contrast to TSCs such as Triapine, with even antagonistic effects with Cu(II).^14,36^ Of note, the stability of the Cu(II) complexes in simple buffered systems is so high (even for Triapine), that their dissociation at pH 7.4 at 1 µM concentration is negligible (<1%).^37^ However, for Triapine derivatives, the Cu(II) complexes can be easily reduced (e.g. with GSH) leading to rapid dissociation, whereas nanomolar-active TSCs show very slow reduction kinetics.^16^ This is most probably also the underlying reason for the different importance of Fe and Cu for the two classes of TSCs. However, how and where TSCs can possibly chelate Cu in the body is widely unknown so far.

The here presented UV-vis and EPR investigations with purified HSA as well as SEC-ICP-MS measurements in human blood serum suggest that the analyzed TSCs Triapine, Dp44mt and Me_2_NNMe_2_ are able to remove Cu(II) from the N-terminal Cu(II)-site of HSA by chelation. In addition, the data revealed that Cu(II)-TSCs can form ternary complexes, most likely with a His on the surface of HSA.^29,30^ Recently, the binding of Cu(II)-Triapine with pure HSA in aqueous 30% DMSO solution was also studied by Enyedy et al.^38^ Moreover, UV-VIS measurements with pure HSA suggest that there is also a non-covalent interaction between the metal-free TSC and binding pocket(s) of HSA.

**Scheme 2:**
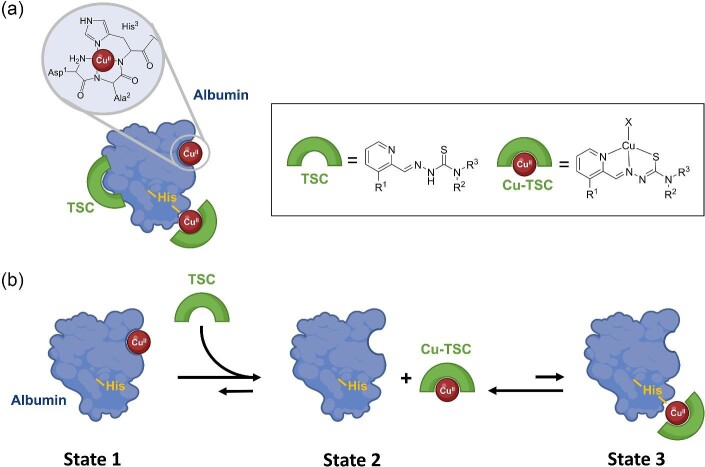
(A) Schematic overview on Cu, TSC, and Cu-TSC binding sites of HSA. (B) Possible equilibria of the interaction of Cu(II)-bearing HSA (state 1) and TSCs, with generation of Cu(II)-TSC (state 2) and its ternary complex with HSA (state 3).

The different possible states and equilibria are depicted in Scheme 2. In state 1, HSA is partially loaded with Cu (<5% of HSA; ∼3 µM Cu-HSA^39^) at the respective *N*-terminal-binding site. Addition of a TSC can result in the removal of the Cu from the HSA-binding site and formation of the respective Cu-TSC complex. In the isolated system at ∼ 10 µM Cu-HSA (Fig. 1), a ternary TSC-Cu-HSA complex is formed (state 3), whereas in serum conditions (Fig. 5) with only ∼3 µM Cu-HSA distinctly lower amounts of Cu-TSC are generated resulting in an equilibrium towards state 2 (free Cu-TSC). In addition, TSCs can also non-covalently bind to HSA (Scheme 2A), which could occur for the excess of TSC added compared to Cu. In state 3, the Cu of the Cu-TSC complex coordinates to a His of the protein forming a ternary complex. Depending on the exact concentrations, these equilibria can also distinctly shift (note that the Cu SEC-ICP-MS analysis of serum cannot distinguish how Cu is bound to HSA, i.e. between state 1 and 3). When pre-formed Cu-TSC (10 µM) is added to the serum, a higher Cu-content at HSA is detected. This increase in Cu content can be attributed to the binding of Cu-TSC to HSA with formation of the ternary complex. The differences in the results of the experiments of adding free TSC alone or Cu-TSC (Fig. 5 vs. Fig. 7), can be explained by an equilibrium shift between states 2 and 3. As described above in serum, only ∼3 µM Cu-HSA are present and addition of 10 µM free TSC only resulted in 2–3 µM Cu-TSC. At this low concentration, the state 2 is predominant. Addition of 10 µM Cu-TSC shifts the equilibrium more to state 3, with a partial formation of the ternary Cu-TSC-HSA complex. Of note, Cu-TSC binds very fast to isolated HSA (within seconds or even faster) but takes several tenth of minutes in serum. Hence, there are other parameters involved that have to be determined in the future (such as a faster binding of Cu-TSC at low concentrations to other proteins and consequently slower equilibration with HSA). This is in contrast to the TSC addition to isolated Cu-HSA and serum, in which the kinetics are consistent between the two systems. Probably a high excess of free TSC with respect to Cu can also influence the binding ability of Cu-TSC to HSA. In general, the ternary binding mode of Cu-TSC complexes with HSA was already shown in X-ray crystal structures by Liang *et al.*, where His146 or His242 was found as the main binding amino acid.^29,30^ Unfortunately, the established SEC-ICP-MS method did not allow for measuring low abundant, non-protein-bound Cu complexes in the low-molecular weight range even with extended measurements up to 40 min, due to poor column recoveries.

These results indicate that TSCs in serum are able to withdraw Cu(II) from HSA, and, hence, can form Cu-TSC complexes. However, the Cu(II) source for TSCs is limited to HSA. Indeed, TSCs were not able to withdraw Cu(II) from the major serum Cu(II) pool ceruloplasmin. The concentration of the ‘accessible’ serum Cu pool is typically about 3 µM,^21,39^ which limits the available Cu to be chelated by TSC. Nevertheless, this pool is a potential source of Cu that could lead to the formation of Cu-TSCs in the human body, which might enter the cells and exert their biological activity. However, it will probably not be able to supply enough copper for all TSC molecules applied as a bolus to the patient. Consequently, additional intracellular Cu sources can probably also be important.

An interesting consideration are the affinities of the three different TSCs to Cu(II), as Dp44mT and Me_2_NNMe_2_ have a distinctly higher stability than Triapine (pCu =−log [Cu(II)] = 11.4 for Triapine; 13.4 for Dp44mT, and 13.3 for Me_2_NNMe_2_).^16^ Consequently, it could be expected that Dp44mT and Me_2_NN_2_Me withdraw Cu(II) better from the *N*-terminal site of HSA than Triapine, which, however, for Me_2_NNMe_2_ seems not to be the case (Fig. 6). However, from the equilibria seen in Scheme 2, the withdrawing of the Cu from HSA depends probably more on the coordination strength of the formed ternary complex of Cu(II)-TSC to His of HSA, than the stability between Cu and TSC.

In conclusion, our data suggest that the labile HSA Cu-pool in serum can act as a, although limited, source of Cu to form Cu-TSC complexes. Hence, it seems important to consider Cu-TSC formation in serum, especially for nanomolar-active TSCs like Dp44mT and Me_2_NNMe_2_, whose Cu-complex also shows higher anticancer activity than the metal-free TSC alone.

## Materials and methods

### Chemicals and reagents

Triapine,^40^ Me_2_NNMe_2_,^36^ and Dp44mT^41^ were prepared as described previously. Ultrapure water (18.2 MΩ cm, ELGA Water purification system, Purelab Ultra MK 2, UK or 18.2 MΩ cm, Milli-Q Advantage, Darmstadt, Germany), nitric acid (≥69%, Rotipuran Supra, Carl Roth, Karlsruhe, Germany), and H_2_O_2_ (30%, Suprapur, Merck, Darmstadt, Germany) were used for the dilutions in the direct infusion ICP-MS measurement. The multielement standard solution, containing 26 elements (Quality Control Standard 26), was purchased from Labkings (Hilversum, The Netherlands). For validation purposes, the certified reference materials TM 28.4, Lake Ontario water, (Environment and Climate change, Burlington, Canada) and Seronorm^TM^, trace elements serum L-1, (Sero AS, Billingstad, Norway) were used. The ammonium acetate buffer applied as eluent for chromatography was derived from ammonium hydroxide solution (25%, Suprapur) and acetic acid (100%, Suprapur), which were purchased by Lactan (Graz, Austria). The protein standards used in the characterization of human serum were all purchased by Athens Research and Technology (Georgia, USA). *N,N*-Dimethylformamide (≥99.9%), Na_2_HPO_4_ (≥99%, EMSURE^®^), NaH_2_PO_4_ (≥99%, EMSURE^®^), human serum (from human male AB plasma, USA origin), and fetal calf serum (USA origin) were all purchased by Sigma Aldrich (Steinheim, Germany).

### Preparation of stock solutions

Cu(II) stock solutions were prepared to dissolve CuCl_2_·2H_2_O in ultrapure water (*ρ* = 18.2 MΩ·cm^−1^). The concentration of CuCl_2_·2H_2_O stock solution was verified by UV-vis spectroscopy (*ε*_780_ = 12 M^−1^cm^−1^). A stock solution of HEPES buffer (500 mM, pH 7.4) was prepared to dissolve free acid powder in ultrapure water and adjusting the pH with NaOH. TSC stock solutions were prepared in DMSO, and their concentration verified via spectrophotometric Cu(II) titrations. HSA stock solution was prepared in ultrapure water and its concentration determined via spectrophotometric Cu(II) titration. Concentrated solutions of Cu(II)-TSC complexes were prepared in DMSO/HEPES (50 mM pH 7.4) 80:20 mixtures.

### UV-vis spectroscopy

UV-vis spectra were recorded in 1 cm path quartz cuvettes using a Cary 60 spectrophotometer equipped with a thermostatted (37°C) multi-cell holder. A 1 µl aliquot of metal-free TSC or pre-formed Cu(II)-TSC complex was added to HSA or to the pre-formed Cu(II)-HSA complex in HEPES 50 mM pH 7.4.

### EPR spectroscopy

CW-EPR spectra were recorded on an EMX-plus (Bruker Biospin GmbH, Germany) X-band EPR spectrometer equipped with a high sensitivity resonator (4119HS-W1, Bruker). The g factor was calibrated in the experimental conditions using the Bruker strong pitch (*g* = 2.0028). The principal experimental parameters values were microwave frequency of ca. 9.4 GHz, microwave power 0.1 mW, modulation amplitude 5 G, time constant of ca. 80 ms, conversion time of ca. 200 ms. Four scans were accumulated to achieve reasonable signal-to-noise (S/N) ratio, resulting in ca. 20 mins of acquisition time per spectrum. Samples were supplemented by 10% v/v glycerol to ensure homogeneous peptide distributions and avoid water crystallization-induced phase separation. Then, they were introduced into 4 mm outer diameter quartz tubes (Wilmad-Labglass) and freeze-quenched into liquid nitrogen prior to their introduction into the precooled cavity (*T* = 100 K, achieved by continuous flow liquid nitrogen cryostat). All experimental EPR spectra were analyzed through computer simulation using homemade scripts based on Easyspin toolbox^42^ environment. Strains on g factor and hyperfine coupling A were used to account for the experimental line broadening.

### Sample preparation for SEC-ICP-MS measurements

#### Buffering of human serum

To keep the pH of human serum at physiological conditions during the incubation with the TSCs, the serum was buffered through the addition of sodium phosphate, 19 mg NaH_2_PO_4_ and 111 mg Na_2_HPO_4_ were mixed with 5 ml of the serum, resulting in a pH of 7.4. The pH value was checked using a pH meter and adjusted accordingly. The serum was analysed by SEC-ICP-MS/MS before and after buffering to ensure that there were no significant changes or contaminations.

#### Incubation with the TSCs

The TSCs were dissolved in minimal amounts of DMF, with 100 µl being sufficient for 1 mg of the respective substances. DMSO was omitted in order to minimize the sulfur background. Further dilutions were prepared using the eluent (50 mM CH_3_COONH_4_) to obtain concentrations ranging from 20 to 2000 µM. After measuring the buffered human serum, 10 µl of these solutions were added to 190-µl serum and mixed thoroughly, resulting in final concentrations of 1–100 µM.

### Instrumentation

#### ICP -MS/MS

An Agilent 8800 ICP-MS/MS instrument (Agilent Technologies, Tokyo, Japan) with oxygen as reaction gas was used to determine the content of the respective elements. The ICP-MS parameters were tuned on a daily based to achieve high sensitivity. The ICP-MS was equipped with a MicroMist nebulizer with a sample uptake rate of ∼0.25 ml·min^−1^ and standard nickel cones. The instrument was coupled to an Agilent 1260 Infinity Bio-Inert HPLC system (Agilent Technologies, Waldbronn, Germany). The Agilent MassHunter software package (Workstation Software, Version C.01.06, 2019) was used for data evaluation. The instrumental parameters for the ICP-MS measurements are summarized in Table 2.

**Table 2.** Instrumental parameters for the ICP-MS/MS measurements using the Agilent 8800

wRF power [W]	1550
Nebulizer	MicroMist
Spray chamber	Scott double-pass
Spraying chamber temperature [°C]	2
Cone materials	Ni
Plasma gas flow [L min^−1^]	15.0
Auxiliary gas flow [L min^−1^]	1.09
Nebulizer gas flow [L min^−1^]	0.90
Measurement mode	O_2_
Reaction gas flow [ml min^−1^]	0.30
Monitored Isotopes	^32^S^16^O, ^56^Fe^16^O, ^63^Cu
Integration time	0.1 s

#### HPLC system

The separation of human serum was carried out using an Acquity UPLC Protein BEH SEC column (1.7 µm, 4.8×300 mm, 200 Å, Waters, Massachusetts, USA). The HPLC parameters are summarized in Table 3.

**Table 3.** Instrumental parameters for the chromatographic separation using the Agilent 1260 HPLC

Autosampler temperature [°C]	37
Injection volume [µl]	5
Column	Acquity UPLC Protein BEH SEC 4.8 × 300 mm, 1.7 µm, 200 Å
Eluent (isocratic)	50 mM CH_3_COONH_4_ pH 6.8
Flow rate [µl min^−1^]	400
Measurement time [min]	12

For the initial SEC-ICP-MS optimization, the following columns were tested to get an ideal separation efficiency (Table 4).

**Table 4.** Parameters for the different columns used in the HPLC optimization process

	Acquity UPLC	Acquity UPLC	Acquity UPLC	Thermo Scientific
Column	Protein BEH SEC	Protein BEH SEC	Protein BEH SEC	MAbPac SEC-1
Dimensions [mm]	4.6 × 150	4.6 × 150	4.6 × 300	4 × 300
Particle size [µm]	1.7	1.7	1.7	5
Pore size [Å]	125	200	200	450
Flow rate [µl min^−1^]	400	400	400	300
Measurement time [min]	8	8	12	20
Column material	Steel	Steel	Steel	PEEK

## Supplementary Material

mfad046_Supplemental_File

## Data Availability

The majority of data underlying this study are available in the manuscript and in the online supplementary material. Additional raw data are available and will be provided by the corresponding author upon reasonable request.
