# Supplementary material for: Human serum albumin as a copper source for anticancer thiosemicarbazones
Source: Metallomics. 2023 Jul 28;15(8):mfad046. doi: 10.1093/mtomcs/mfad046 (PMC10405564; doi:10.1093/mtomcs/mfad046)
Supplement: mfad046_Supplemental_File [file mfad046_supplemental_file.docx]

**SUPPORTING INFORMATION**

**Human Serum Albumin as a Copper Source for Anticancer Thiosemicarbazones**

Martin Schaier,^1,2¥^ Enrico Falcone,^3¥^ Tomas Prstek,^1^ Bertrand Vileno^3^, Sonja Hager,^4,5^ Bernhard K. Keppler,^6,7^ Petra Heffeter,^4,7*^ Gunda Koellensperger,^1,7*^ Peter Faller^3,8*^, Christian R. Kowol^6,7*^

^1^ Institute of Analytical Chemistry, Faculty of Chemistry, University of Vienna, Waehringer Str. 38, A-1090 Vienna, Austria

^2^ Vienna Doctoral School in Chemistry (DoSChem), University of Vienna, Waehringer Str. 42, A-1090 Vienna, Austria

^3^ Institut de Chimie, UMR 7177, CNRS, Université de Strasbourg, 4 Rue Blaise Pascal, 67000 Strasbourg, France

^4^ Center for Cancer Research and Comprehensive Cancer Center, Medical University of Vienna, Borschkegasse 8a, A-1090 Vienna, Austria

^5^ Institute of Food Chemistry and Toxicology, Faculty of Chemistry, University of Vienna, Waehringer Str. 38, A-1090 Vienna, Austria

^6^ Institute of Inorganic Chemistry, Faculty of Chemistry, University of Vienna, Waehringer Str. 42, A-1090 Vienna, Austria

^7^ Research Cluster ‘Translational Cancer Therapy Research’, A-1090 Vienna, Austria

^8^ Institut Universitaire de France (IUF), 1 rue Descartes, 75231 Paris, France.

^¥^ These authors contributed equally to the main findings of this manuscript.


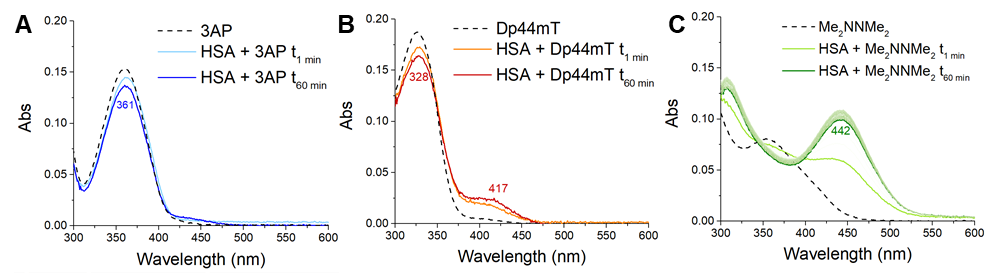

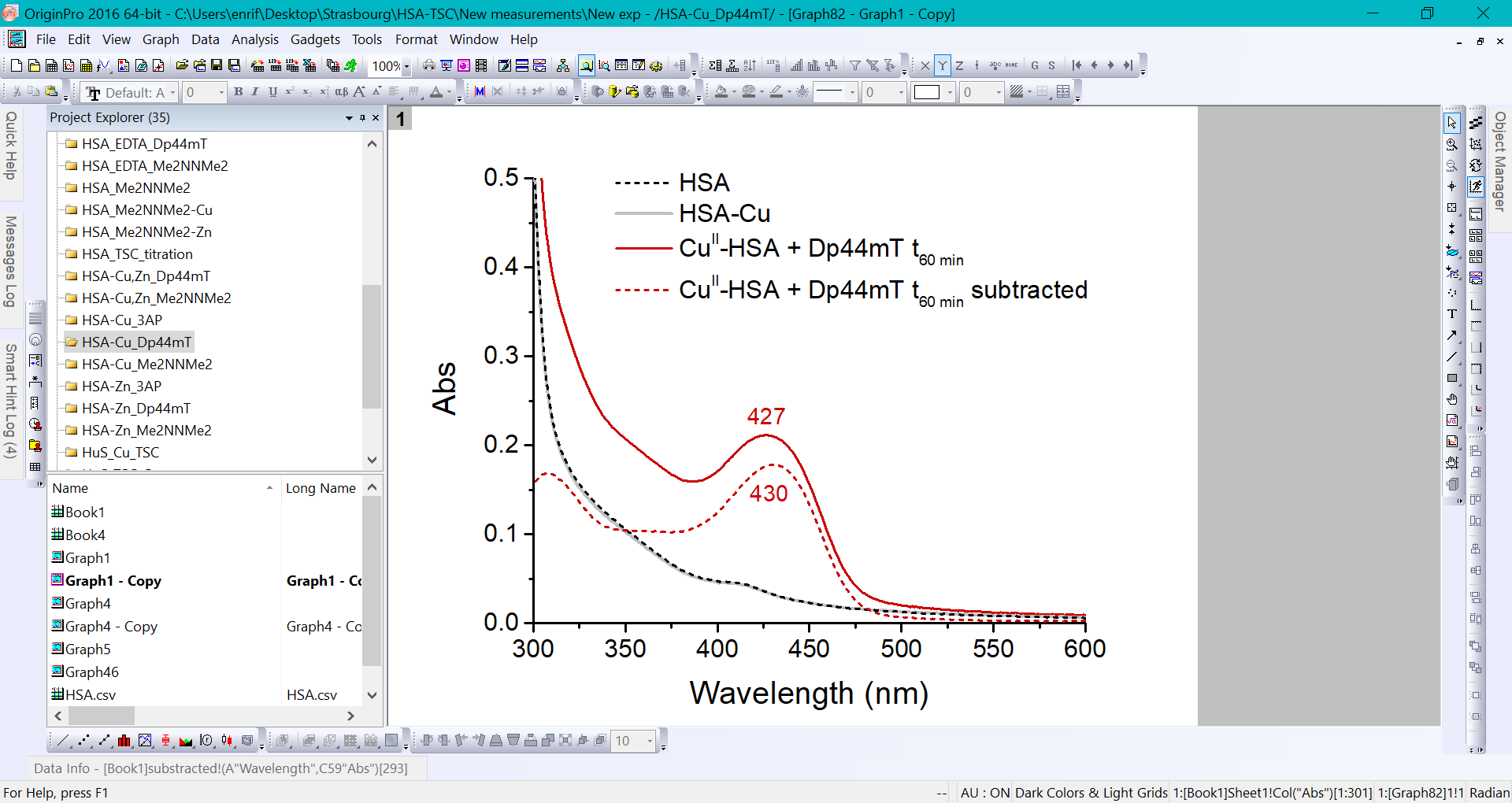


**D**

**Figure S1.** Interaction of TSCs with HSA. A) Triapine (blue); B) Dp44mT (red); C) Me_2_NNMe_2_ (green). Conditions: [TSC] = 10 μM, [HSA] = 100 μM, HEPES 50 mM pH 7.4 (DMSO 1%). T = 37 °C. D) Example for the HSA/HSA-Cu background subtraction performed for the spectra in Figure 1 and S1.

**
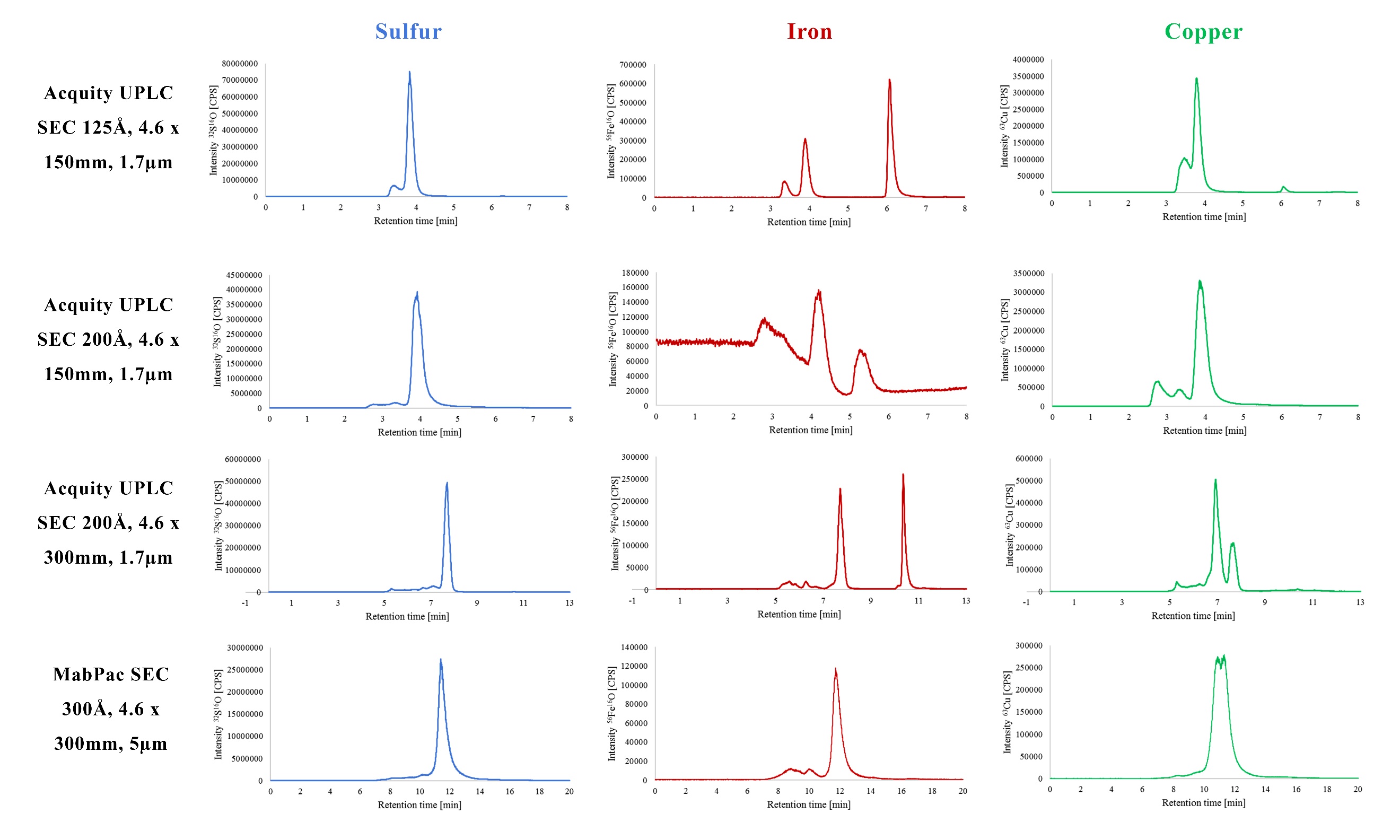
**

**Figure S2:** HPLC-ICP-MS measurements of sulfur, iron and copper in human serum using different size exclusion columns.


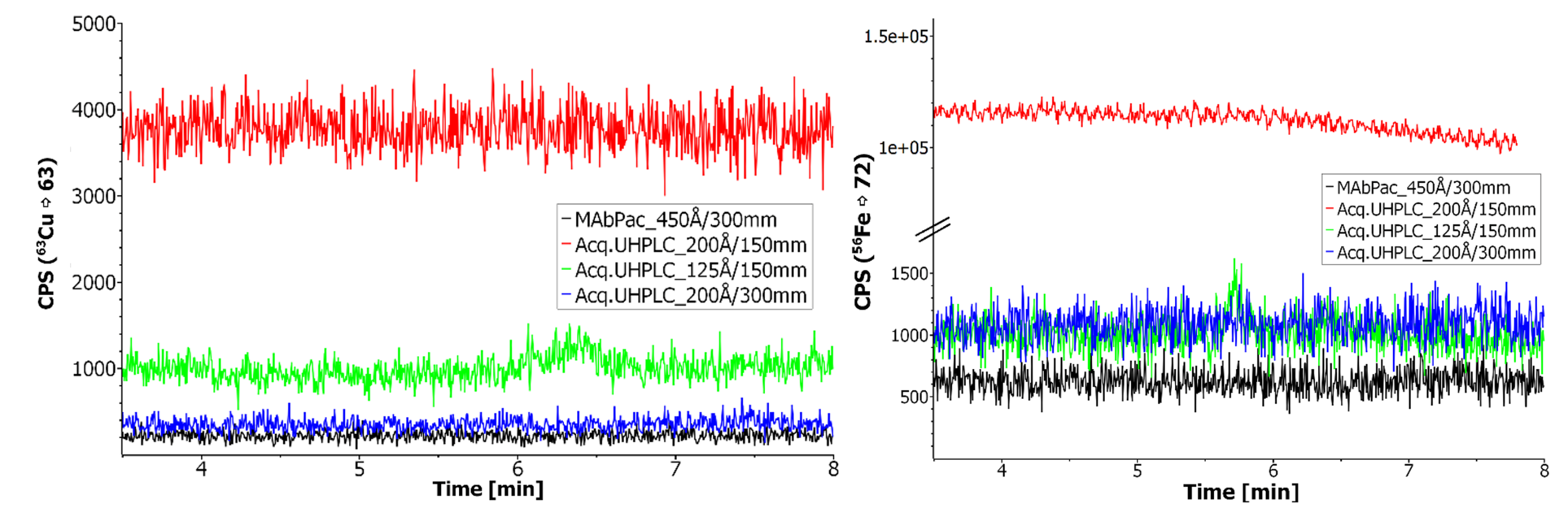


**Figure S3:** Evaluation of Cu and Fe background for the different columns (high background in the case of Acquity UPLC SEC 200 Å/150 mm is most likely due to extensive previous use).

**
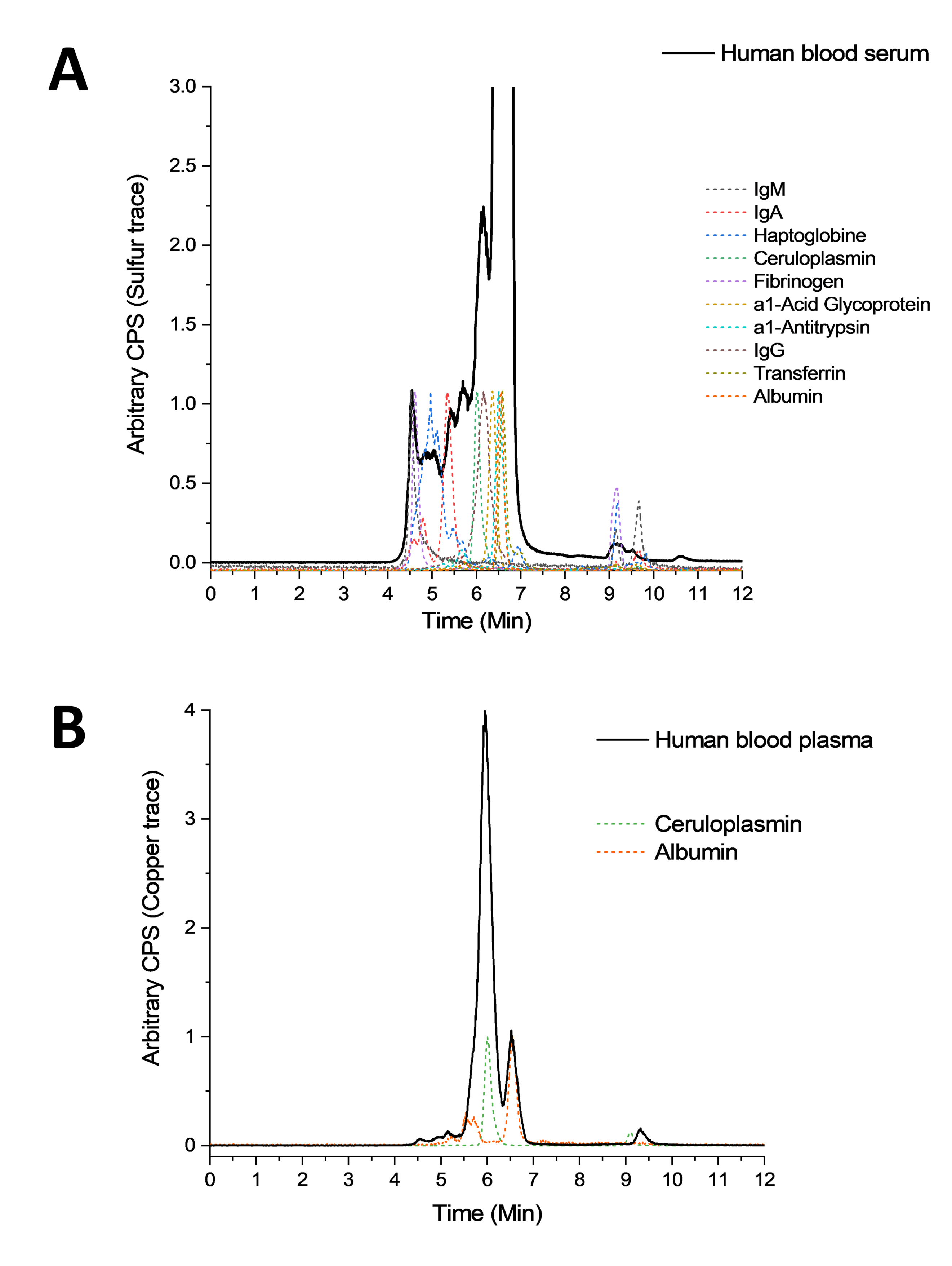
**

**Figure S4:** A) Overlay plot of the SEC-ICP-MS sulfur trace chromatogram (0.0–12.0 min) of human blood serum (black solid line) with 10 protein standards (dotted lines). B) Overlay plot of the whole ICP-MS copper trace of human blood serum chromatogram (black solid line) with albumin and ceruloplasmin (dotted lines).


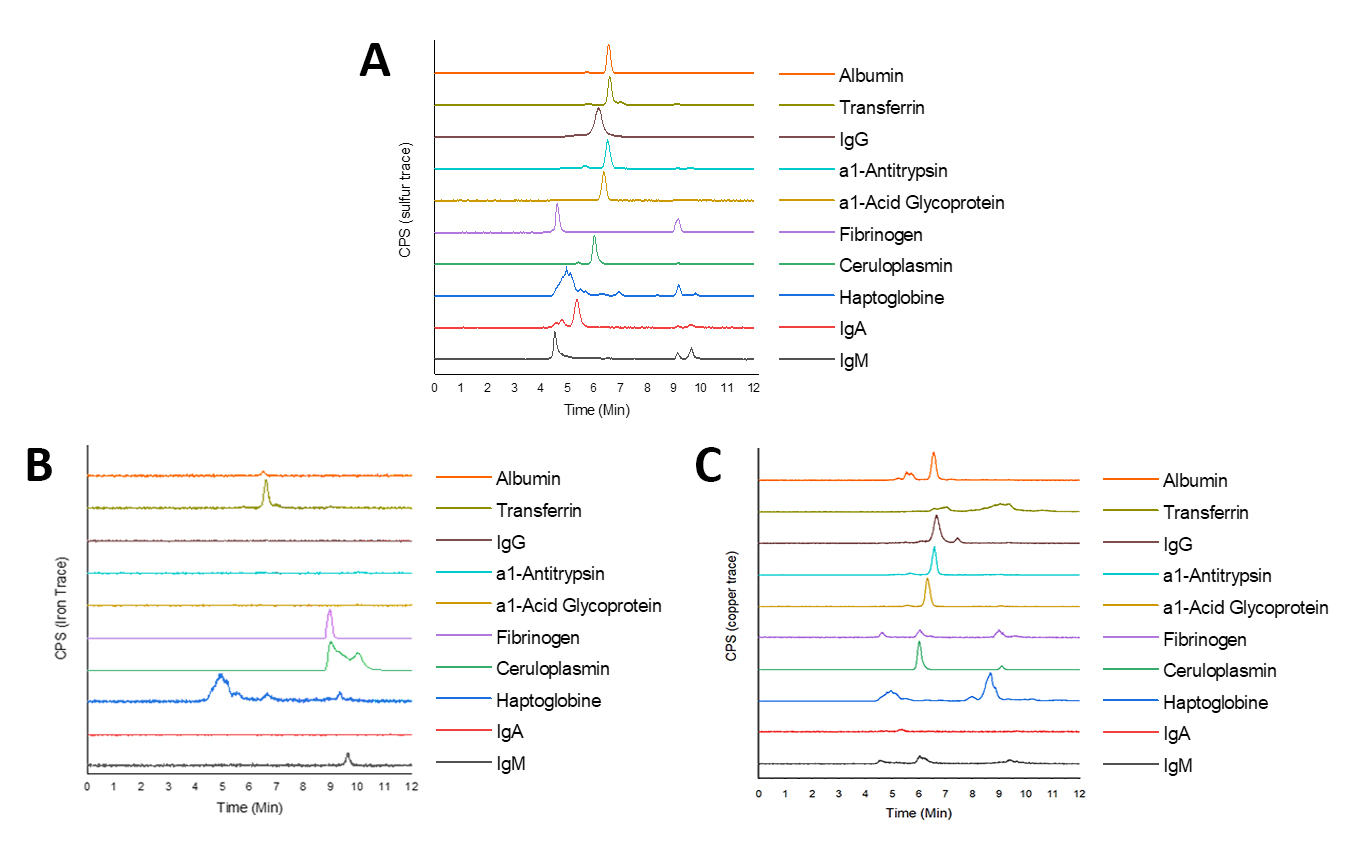


**Figure S5.** Stacked chromatograms for A) sulfur, B) iron, and C) copper traces for the 10 protein standards measured by SEC-ICP-MS.


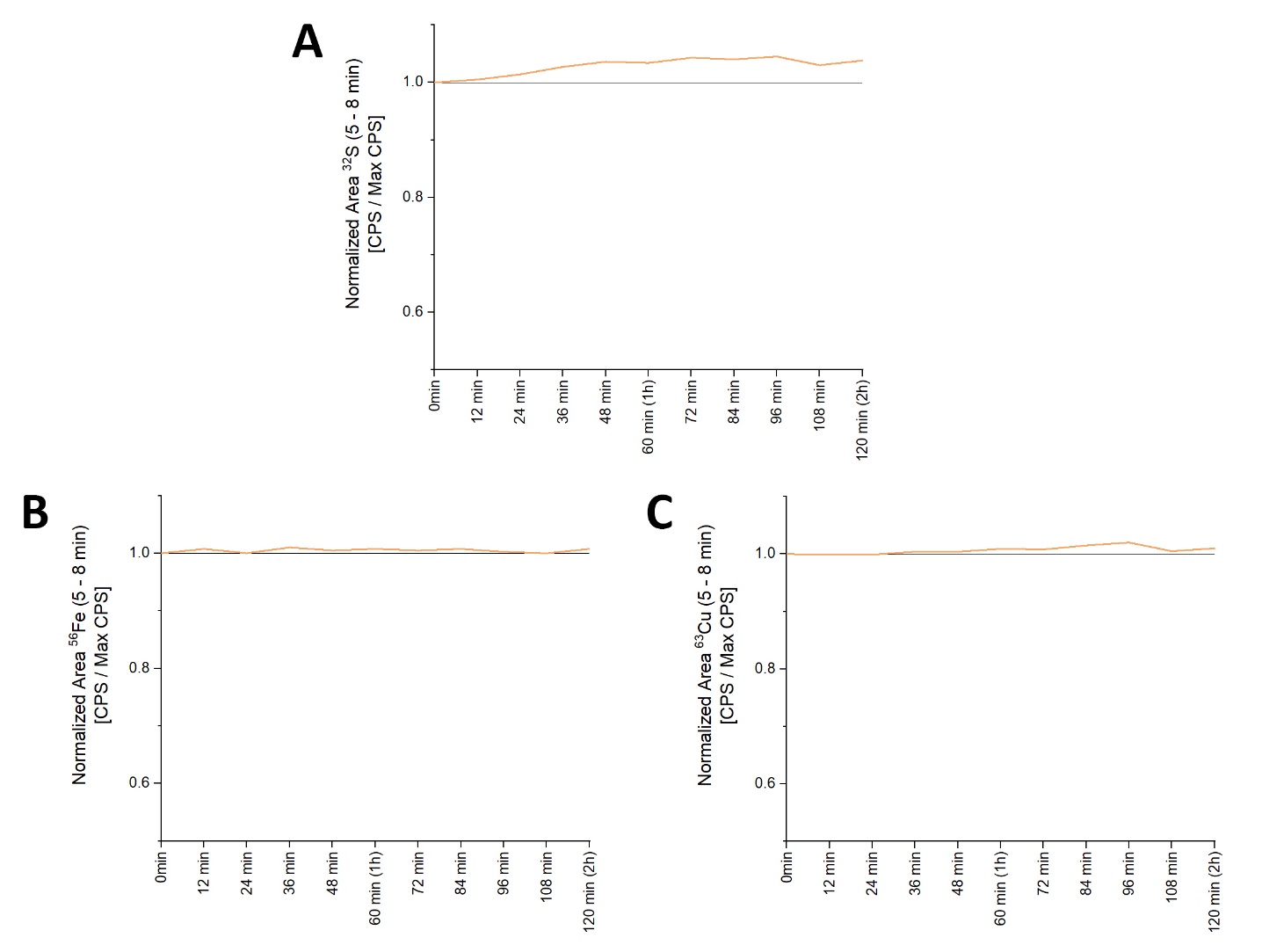


**Figure S6:** A) Sulfur, B) iron, and C) copper signal stability for human serum over 120 min measured with SEC-ICP-MS.


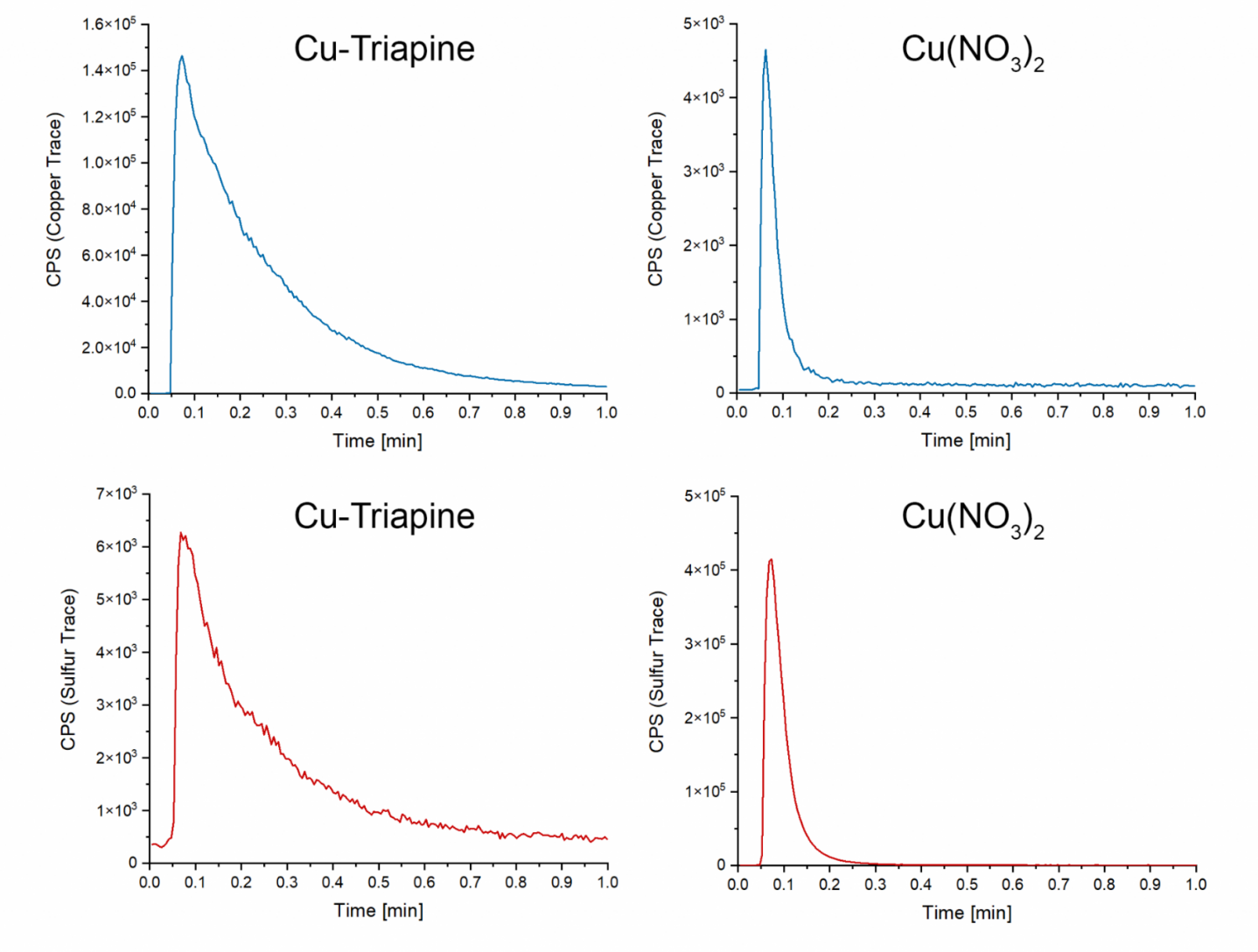


**Figure S7:** Elution behavior of Cu-Triapine compared to a standard solution of Cu(NO_3_)_2_ in ICP-MS flow injection analysis (sulfur and copper trace).

**Figure S8:** Copper traces of human serum treated with different metal-free TSCs (10 µM) for 120 minutes, measured by SEC-ICP-MS. The first chromatogram is untreated serum as a control.

**Figure S9:** Iron traces of human serum treated with different metal-free TSCs (10 µM) for 120 minutes, measured by SEC-ICP-MS. The first chromatogram is untreated serum as a control.

**Figure S10:** Copper traces of human serum treated with different Cu-TSCs (10 µM) for 120 minutes, measured by SEC-ICP-MS. The first chromatogram is untreated serum as a control.

**Table S1.** EPR parameters (*g*_//_ and A_//_) of the spectra reported in Figure 2.

| **Complex** | ***g*_//_** | **A_//_ (MHz)** |
| --- | --- | --- |
| Cu-HSA | 2.186 | 600 |
| Cu-Dp44mT | 2.196 | 539 |
| HSA-Cu-Dp44mT | 2.178 | 555 |
| Cu-Dp44mT-Im | 2.182 | 565 |
| Cu-3AP | 2.200 | 547 |
| HSA-Cu-3AP | 2.180 | 555 |
| Cu-3AP-Im | 2.188 | 535 |
| Cu-Me_2_NNMe_2_ | 2.194 | 545 |
| HSA-Cu-Me_2_NNMe_2_ | 2.182 | 550 |
| Cu-Me_2_NNMe_2_-Im | 2.184 | 548 |
| GSH-Cu-Dp44mT | 2.141 | 532 |
